# Synthesis and Nanoprecipitation of HEMA-CL*_n_* Based Polymers for the Production of Biodegradable Nanoparticles

**DOI:** 10.3390/polym9090389

**Published:** 2017-08-23

**Authors:** Simone Gatti, Azzurra Agostini, Raffaele Ferrari, Davide Moscatelli

**Affiliations:** 1 Department of Chemistry, Materials and Chemical Engineering “Giulio Natta”, Politecnico di Milano, via Mancinelli 7, 20131 Milan, Italy; simone.gatti@polimi.it (S.G.); azzurra.agostini@polimi.it (A.A.); 2 Institute for Chemical and Bioengineering, Department of Chemistry and Applied Biosciences, ETH Zürich, Vladimir-Prelog-Weg 1, 8093 Zürich, Switzerland; raffaeleferrari86@gmail.com

**Keywords:** RAFT polymerization, nanoparticles, poly(ε-caprolactone), nanoprecipitation, degradation

## Abstract

The control over the size distribution and stability of polymeric nanoparticles (NPs) is crucial in many of their applications, especially in the biomedical field. These characteristics are typically influenced by the production method and the nature of the starting material. To investigate these aspects, the controlled radical polymerization of functionalized methacrylates constituted by 2-hydroxyethyl methacrylate (HEMA) functionalized with a controlled number of ε-caprolactone (CL) units (HEMA-CL*_n_*), was carried out via reversible addition–fragmentation chain transfer polymerization (RAFT) in solution. The living reaction allows for good control over the molar mass of the final polymer with a low molar mass dispersity. The obtained polymer solutions were nanoprecipitated in order to produce NPs suitable for drug delivery applications with narrow particle size distribution and a wide size range (from 60 to 250 nm). The NP synthesis has been performed using a mixing device, in order to control the parameters involved in the nanoprecipitation process. As already seen for similar systems, the size of the produced NPs is a function of the polymer concentration during the nanoprecipitation process. Nevertheless, when the polymer concentration is kept constant, the NP size is influenced by the chemical structure of the polymer used, in terms of the presence of PEG (poly(ethylene glycol)), the degree of RAFT polymerization, and the length of the caprolactone side chain. These characteristics were also found to influence the stability and degradation properties of the produced NPs.

## 1. Introduction

Over the last decades, interest in developing biodegradable polymeric nanoparticles (NPs) as effective drug delivery systems has increased [1,2,3]. The purpose is the creation of a drug vehicle that allows the slow release of the drug (i.e., temporal control) [4], or carries the drug to the site of activity (i.e., distribution control) [5], in order to improve the effectiveness of the drug and to decrease side effects.

In designing those systems, it is very important to have a good control over the chemical structure of the polymer, and in particular over its molar mass. This control allows for a fine tuning of the nanocarrier characteristics, such as dimension, lipophilicity and degradation time [6,7]. These characteristics of the nanocarrier also influence the encapsulation efficiency and release profile of encapsulated drugs, the biodistribution of the NPs, their toxicity, and their ability to be efficiently excreted from a living organism. 

The discovery of new techniques to perform controlled/living radical polymerization (CRP) has enabled the synthesis of a variety of macromolecular systems with controlled structure, molar mass, and narrow molar mass distribution [8,9]. Among the various techniques used to control radical polymerization, the most studied and adopted are nitroxide-mediated polymerization (NMP) [10,11], atom transfer radical polymerization (ATRP) [12,13], and reversible addition–fragmentation chain transfer polymerization (RAFT) [14,15,16]. Each of these methods has advantages as well as limitations. However, in this work RAFT polymerization has been selected, since this technique enables control of the polymerization reactions only through adding a chain transfer agent and without using toxic compounds, such as metal catalysts [17]. Moreover, due to its tolerance to a wide variety of solvents, temperatures, monomers, and functional groups [18], the RAFT polymerization can be easily adopted for the production of biocompatible polymers. 

**Scheme 1:**
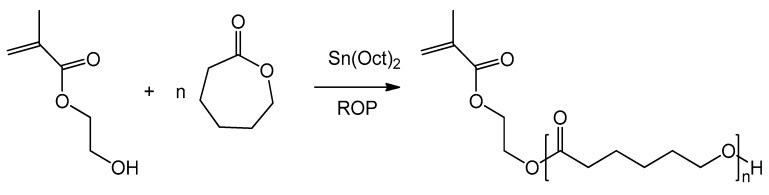
Production of the 2-hydroxyethyl methacrylate ε-caprolactone (HEMA-CL*_n_*) macromonomer via ring opening polymerization (ROP) reaction.

Recent works [19,20] have presented a new type of functionalized monomers based on 2-hydroxyethyl methacrylate (HEMA) functionalized with a tunable number of ε-caprolactone (CL) units. The synthesis of these HEMA-CL*_n_* macromonomers (Scheme 1) is carried out through ring opening polymerization (ROP) [21,22]. Due to the living character of this reaction, it is possible to control the number of caprolactone units added to the HEMA molecule (*n*). It is also possible to produce macromonomers with different side chains, made for example of poly(lactic acid) or poly[(lactic acid)-*co*-(glycolic acid)], simply by changing the cyclic ester in the ROP synthesis [23,24].

In previous works, these macromonomers were copolymerized with commercially available PEG-based macromonomers, such as poly(ethylene glycol) methyl ether methacrylate (PEGMA), through emulsion-free radical polymerization in order to produce biodegradable PEGylated NPs [25]. These NPs were successfully applied to drug delivery applications; for example, they were loaded with hydrophobic drugs (minocycline and paclitaxel) and used in the treatment of spinal cord injury [26], or cancer [27]. Emulsion radical polymerization allows the researcher to obtain a very good control over particle size, but not a good control over the *M*_n_ of the polymer. Moreover, since NP formation occurs during the polymerization in this process, it is not possible to encapsulate a hydrophobic drug inside the NP core during the synthesis. The drug is usually added on the NP surface through nanoprecipitation using preformed NPs, leading to low drug loading and fast drug release. 

**Scheme 2:**
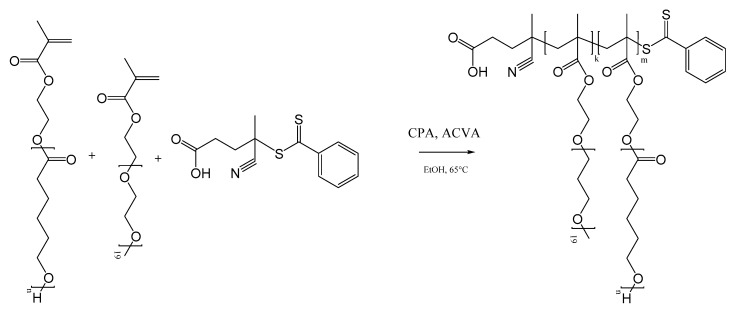
Random reversible addition–fragmentation chain transfer polymerization (RAFT) copolymerization of HEMA-CL*_n_* and poly(ethylene glycol) methyl ether methacrylate (PEGMA).

In order to overcome these issues, in this work RAFT homopolymerization of the hydrophobic HEMA-CL*_n_* was applied to control the molar mass of the final polymer, which is then nanoprecipitated to produce NPs. In this way, we can obtain a comb-like polymer structure where it is possible to tune both the number of CL units on the side chains (*n*), and the number of HEMA-CL*_n_* units (*m*), as reported in Scheme 2. Moreover, in order to produce NPs with stable behavior in biologically relevant fluids, the HEMA-CL*_n_* macromonomers were also copolymerized with a small quantity of a PEGMA, which was added in the polymerization step to produce a random comb-like copolymer with both hydrophobic and hydrophilic chains (indicated with the letter *k*). The obtained polymer structure is reported in Scheme 2.

Macromonomers HEMA-CL*_n_*, with *n* equal to 2, 3 and 5, were produced and characterized as already described [28], and then polymerized in ethanol through RAFT polymerization using 4-Cyano-4-(phenylcarbonothioylthio)pentanoic acid (CPA), a RAFT agent that has been proven as effective for the polymerization of other methacrylates [29]. Characterization via gel permeation chromatography (GPC) and nuclear magnetic resonance (NMR) analysis proved the polymer formation and the living character of the polymerization reaction of these macromonomers. Since these NPs are intended for biological applications, the synthesis was carried out in ethanol, which was chosen as a suitable solvent that allows the avoidance of intensive purification of the final product. The polymer solution has been then used to produce nanoparticles exploiting an effective, low-cost, easy to scale-up microfluidic device, and to study the influence of the process parameters, such as the concentration of the polymer solution as well as the polymer characteristics. The NPs produced were then characterized via dynamic light scattering (DLS) in order to investigate the effect of the polymer structure on the particle size distribution, the stability and the NP degradation properties.

## 2. Materials and Methods 

### 2.1. Materials

ε-Caprolactone (CL, Acros, 99% purity), 2-ethylhexanoic acid tin(II) salt (Sn(Oct)_2_; Sigma-Aldrich, St. Louis, MO, USA, purity ≥95%), 4,4′-Azobis(4-cyanovaleric acid) (ACVA; Sigma-Aldrich, purity ≥ 98%), 4-Cyano-4-(phenylcarbonothioylthio)pentanoic acid (CPA; Sigma-Aldrich, purity ≥ 97%), poly(ethylene glycol) methyl ether methacrylate (PEGMA; Sigma-Aldrich, *M*_n_ 950 Da), Tween80 (Sigma-Aldrich), Diethyl ether (DEE, Sigma-Aldrich, ≥99%), Deuterated chloroform (CDCl_3_, Sigma-Aldrich, 99.8%), Ethanol (EtOH, Sigma-Aldrich, 99.8%), acetonitrile (ACN, Sigma-Aldrich, 99.9%), 6-hydroxycaproic acid (HCA, ABCR, Karlsruhe, Germany, 95%) were used as received without further treatments. The cell medium is composed by high glucose DMEM/F12 (Biowest, Nuaillé, France) supplemented with 10% fetal bovine serum (Lonza, Basel, Switzerland). Phosphate-buffered saline solution (PBS) was purchased from Sigma-Aldrich. For gel permeation chromatography (SEC) analysis Tetrahydrofuran (THF, Sigma-Aldrich, purity ≥ 99.7%) was used as eluent. 

### 2.2. Polymer Synthesis

Three macromonomers were synthesized (HEMA-CL_2_, HEMA-CL_3_ and HEMA-CL_5_) by changing the molar ratio between CL and HEMA, as already described in the literature [19]. Briefly, for the case of HEMA-CL_3_, Sn(Oct)_2_ (30 mg, 0.074 mmol) was mixed with HEMA (3.8 g, 0.029 mol) in a 10-mL vial under magnetic stirring at room temperature until full dissolution of the Sn(Oct)_2_. Meanwhile, CL (10 g, 0.0877 mol) was heated up to 130 °C in a stirred round-bottom flask. The HEMA/Sn(Oct)_2_ solution was then added to the flask, and the reaction was carried out for 2 h. Characterization was carried out via ^1^H NMR analysis performed in CDCl_3_; details can be found in the Supplementary Materials, Figure S1.

All the HEMA-CL*_n_* polymerizations were carried out using ethanol as solvent, CPA as the RAFT agent, and ACVA as the radical initiator. The degree of polymerization (DP) was controlled by changing the molar ratio between the monomers and the RAFT agent. Two classes of polymers were produced. In the first class, the HEMA-CL*_n_* monomers were homopolymerized, which changed the length of the CL side chain (*n*) and the number of HEMA-CL*_n_* units in the polymer molecule (*m*). The sample names for the first class of polymers are reported as *m*CL*_n_*. In the second class of polymers, HEMA-CL*_n_* monomers were copolymerized with PEGMA (10% mol respect with HEMA-CL*_n_*), which changed the length of the CL side chain (*n*) and the number of HEMA-CL*_n_* units in the polymer molecule (*m*). The sample names for the second class of polymers are reported as *m*CL*_n_*PEG.

An example of these syntheses is reported for the sample 20CL_3_PEG: HEMA-CL_3_ (1 g, 2.11 mmol), PEGMA (0.21 g, 0.21 mmol), CPA (30 mg, 0.106 mmol), ACVA (9.84 mg, 0.0352 mmol) and EtOH (2.73 g, 59.3 mmol) were mixed in a round-bottom flask. The mixture was purged with nitrogen for 10 min to remove the oxygen, and then heated at 65 °C in an oil bath for 24 h. A sample of the reaction mixture was then dried under vacuum and dissolved in CDCl_3_ and in THF for the ^1^H NMR and GPC characterizations. The samples were then evaporated and precipitated in DEE twice.

The monomer conversion was determined by ^1^H NMR, using a 500 MHz Ultrashield NMR spectrometer (Bruker, Billerica, MA, USA) by comparing the peaks relative to the double bond and a reference peak, as reported in the Supplementary Materials, Figure S2. The number-average molar mass (*M*_n_) and molar mass dispersity (*M*_w_/*M*_n_) of the polymers were obtained via gel permeation chromatography (GPC) using THF as eluent at a flow rate of 0.5 mL/min and a column temperature of 35 °C. Samples were dissolved in THF at a concentration of 4 mg·mL^−1^ after filtration through a 0.45 μm pore-size membrane, and analyzed with a Jasco (Series) apparatus (Oklahoma City, OK, USA) equipped with a differential refractive index (RI) detector. The separation was performed at a flow rate of 0.5 mL·min^−1^ and at 35 °C with three Superchrom PLgel 5 μm columns (600 × 7.5 mm, measuring range 0.5–1000 kDa). Universal calibration based on polystyrene standards was applied.

### 2.3. Nanoparticle Production, Stability and Degradation Studies

**Figure 1:**
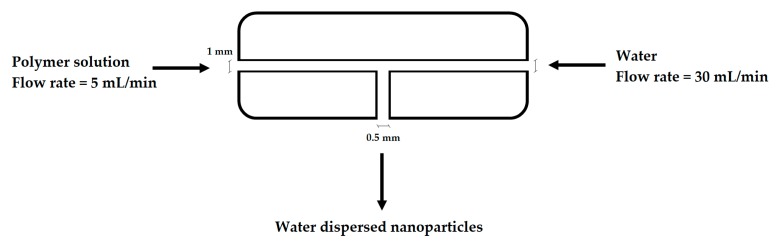
Mixing device schematization.

The NP synthesis has been performed using a mixing device in order to finely control the parameters involved in the nanoprecipitation process and thus obtaining a good reproducibility. This device is constituted by a T-mixing PTFE cylinder of 1 cm of diameter and 1 cm of length, with an axial perforation of 1 mm diameter and a radial one of 500 μm. As described in Figure 1, water and an ethanol solution of the polymer were loaded in syringe pumps and injected radially in the mixing device at a flow rate of 30 mL·min^−1^ and 5 mL·min^−1^, respectively. The non-PEGylated polymers were nanoprecipitated both in pure water and in a solution 0.1 wt % of Tween80 in water. When PEGylated polymers were employed in the NP synthesis, just purified water was used.

After the synthesis, NPs were characterized in terms of average diameter (NP size) and polydispersity index (PDI) by dynamic light scattering (Malvern Zetanano ZS, Malvern, UK) using the cumulant method, as defined by ISO (standard document 13321:1996E); all the reported data are an average between two measures of the same sample.

The produced NPs have been employed in further studies to evaluate their stability and degradation in biologically relevant fluids: cell medium and PBS (10 mM, pH 7.4). 1 mL of NP suspension (with a polymer concentration of 0.1% *w/w*) was diluted in 1 mL of the selected medium and maintained in a heating oven at 37.0 ± 0.1 °C. The evolution of size particle and particle size distribution of the samples was studied through dynamic light scattering (DLS) measurements.

The degradation of the sample 20CL3PEG was further investigated via HPLC. The polymer was nanoprecipitated as previously, with a final polymer concentration of 5 mg/mL. 10 mL of NP suspension was added to 10 mL of three different solutions: PBS, a cell medium, and a lipase solution (2 mg/mL of lipase from porcine pancreas in PBS), and incubated at 37 °C. Aliquots of 500 µL were withdrawn at different time points and ultrafiltrated with vivaspin500 10 kDa MWCO. The filtered solution was analyzed by an HPLC apparatus (Agilent 1100 series, Santa Clara, CA, USA) equipped with a UV–Visible spectrometer detector and a mass spectrometer detector (Agilent 6140 quadrupole LC/MS, Santa Clara, CA, USA). The samples were eluted using water (0.1% formic acid) and acetonitrile (0.1% formic acid). The concentration of the eluent was changed from 3% to 100% acetonitrile over 100 min (gradient from 3% to 20% for 10 min, isocratic at 20% for 20 min, gradient from 20% to 100% for 60 min, pure acetonitrile for 10 min). A calibration based on UV absorption was made with 6-hydroxycaproic acid (HCA) in a concentration range from 0.1 to 10 mg/mL. The identification of the peaks was supported by the MS detector. The % of degradation was calculated with the following equation:

(1) $$\%Degradation=\frac{C_{HCA}}{C_{max}}\times 100$$

where *C*_HCA_ is the concentration of HCA in the samples, obtained by HPLC, and *C*_max_ is the maximum concentration of 6-hydroxycaproic acid that the sample can theoretically reach after the complete degradation, computed by taking into account the concentration of the polymer and the content of caprolactone in the polymer. 

## 3. Results

### 3.1. Polymer Production and Characterization

**Figure 2:**
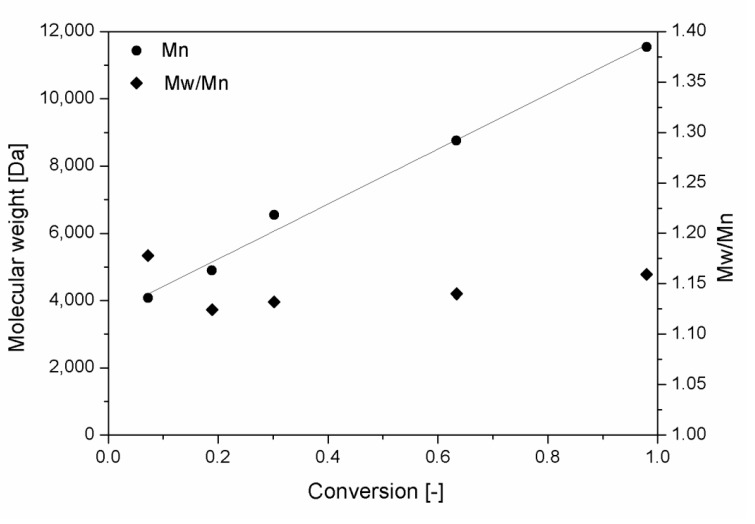
RAFT polymerization of 20CL_3_. Molar mass and molar mass dispersity as a function of the macromonomer conversion.

In this study, the RAFT polymerization of three ε-caprolactone based macromonomers was investigated, in order to understand the different behavior of polymers in the nanoprecipitation process. As already reported, for every polymerization a stated value of the [M]_0_/[CTA]_0_ ratio was chosen ([M]_0_ is the initial concentration of the monomer, [CTA]_0_ is the initial concentration of the RAFT agent), which corresponded to a target DP. To prove the livingness of the process, in Figure 2 the *M*_n_ of the polymer for the polymerization of a HEMA-CL_3_ macromonomer, with a target DP equal to 20 (20CL_3_), has been plotted in function of the conversion. A good linearity of the *M*_n_ of the polymer over the conversion is found as expected from Equation (2).

(2) $$M_{n}^{\left(theoretical\right)}=\frac{{\left[M\right]}_{0}}{{\left[CTA\right]}_{0}}\chi M_{mon}+M_{CTA}$$

where *M*_mon_ is the molar mass of the monomer, χ is the monomer conversion, and *M*_CTA_ is the molar mass of the RAFT agent. As the monomer conversion increases, the retention time of the samples injected in the GPC decreases, and thus, the *M*_n_ increases. The molar mass distribution remains narrow, as shown from the low value of the final *M*_w_/*M*_n_ reported in Table 1. This suggests the low impact of the bimolecular combination of active chains, and a prominent presence of dormant chains that could still grow when another quantity of the same monomer is added. This also suggests the possibility of other post-modifications, such as the production of block copolymers [30,31] or bioconjugation [32]. In Figure 2, we also observe a deviation from the latter equation at a low value of conversion, probably due to a slow re-initiation of the radical released from the RAFT agent [33]. As a controlled polymerization, the molar mass dispersity remains low during the whole reaction time (Figure 2). 

In Table 1, polymer characteristics are shown for different macromonomer polymerizations in ethanol using CPA as the chain transfer agent and ACVA as the initiator at 65 °C for 24 h. Polymerizations were carried out with different initial molar ratios [M]_0_/[CTA]_0_ (m) and using macromonomers with different side chain lengths (*n*). For the PEGylated polymers, PEGMA (10% mol respect to HEMA-CL*_n_*) was also added in the reaction mixture. For these samples, the composition of the produced polymer was verified by ^1^H NMR (Figure S2). For all the reactions, a good conversion (χ > 0.9) was reached and the resulted polymers showed low molar mass dispersity (*M*_w_/*M*_n_ < 1.2). A good correspondence between the average *M*_n_ obtained from GPC and its theoretical value was found, again confirming the good control of the RAFT reaction over the polymerization process. 

### 3.2. NP Production and Characterization

**Figure 3:**
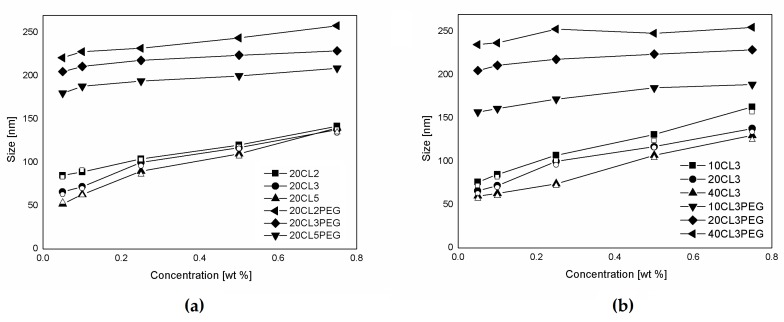
NPs produced via nanoprecipitation with different polymer samples as a function of the polymer concentration. (**a**) NPs produced at constant DP (*m* = 20) with polymer samples synthesized with different macromonomers (*n* = 2, 3, 5). (**b**) NPs produced at constant side chain ε-caprolactone (CL) length (*n* = 3) with polymer samples with different backbone length (*m* = 10, 20, 40). The hollow symbols represent the non-PEGylated polymers, nanoprecipitated without using surfactant.

The produced polymer samples were dissolved in ethanol and used to produce NPs via a nanoprecipitation process, as already described. The solutions were prepared in order to obtain a final polymer concentration in the water suspension between 0.1 and 0.75% *w/w*. In Figure 3, the NP sizes obtained from the nanoprecipitation of the polymers are shown for different values of the polymer concentration. The non-PEGylated polymers were nanoprecipitated in both pure water and a solution 0.1% of Tween80 in water, but in these conditions, the presence of the surfactant does not influence the size of the produced NPs. With a constant theoretical DP equal to 20 (Figure 3a), the NP size is mainly determined by the hydrophobicity of the polymer used, which is a function of the macromonomer type and the polymer *M*_n_. Accordingly, due to their higher hydrophilicity, PEGylated samples produce bigger NPs than the corresponding non-PEGylate ones. This behavior was already observed in other works concerning NPs produced via emulsion polymerization of these biodegradable macromonomers [34]. Another aspect investigated here is the influence of the polymer concentration on the NP production. For the non-PEGylated samples, the NP size is strongly dependent on the polymer concentration. As the polymer concentration increases, the NP size increases with a linear correlation. This behavior is due to the nature of the nanoprecipitation process [35,36]. If the fluid dynamic behavior of the mixing is constant, as it is in this case due to the fine control over the process parameters allowed by the use of the microfluidic device, the concentration of particles formed in the nucleation process is to be considered constant. However, while ethanol diffuses in water, nanoparticles increase their size in relation to the amount of polymer that surrounds them, and then as a function of the polymer concentration in the ethanol solution. The slope of this function is lower for the PEGylated samples in which the NP size seems to be less influenced by the polymer concentration and more influenced by the structure of the polymer. In Figure 3b, the NP size obtained as a function of the polymer concentration and the DP is reported. In particular, two different behaviors are observed for PEGylated and non-PEGylated particles. For the non-PEGylated samples, a higher DP leads to a lower NP size. This is probably due to the effect of the polymer hydrophobicity, since a hydrophobic polymer with a higher *M*_n_ tends to form more compact and less swelled NPs. This fact is consistent with the known property of hydrophobic polymers, in which their hydrophobicity increases with the *M*_n_. For the PEGylated samples, a higher *M*_n_ leads to a higher NP size. This behavior seems to be more related to the configuration of the polymer that forms the NPs. Since the PEG side chains are covalently linked to the polymer structure, the NPs will be stabilized by the hydrophilic PEG chains without the use of surfactants, such as an amphiphilic block copolymer. A similar behavior can be found in another work, in which block copolymers made of PEGMA and methyl methacrylate (MMA) were polymerized via RAFT emulsion polymerization, and a linear correlation between the DP of the hydrophobic portion was found [37]. In this case, the structure of the copolymer is random, or moderately gradient, since the two monomer’s reactivity can be slightly different [38]. Either way, the configuration of the polymer in the NPs is less ordered and the NP size also depends on the polymer concentration. Nevertheless, a positive correlation between the polymer DP and the NP size is observed.

Finally, the NP characterization obtained by DLS is reported in Table 2. The produced NPs shows a wide size range between 63–237 nm, which is compatible for biomedical applications such as intravenous drug delivery. Moreover, NPs are monodispersed, and the polydispersity index (PDI) is very low for all the samples produced.

### 3.3. NP Stability and Degradation Behavior

The NPs produced with the nanoprecipitation process were used for further stability and degradation studies in different fluids (cell medium and PBS) at 37 °C to evaluate their colloidal and chemical stability in media of interest, and to make preliminary predictions about their behavior in biological environments. Cell medium is an aqueous mixture of high glucose DMEM/F12 and fetal bovine serum. It is frequently used to grow the cells employed for in vitro experiments typical of the biological evaluation of NPs such as cytotoxicity and cellular uptake experiments. It is also a protein-rich medium, since it contains a high amount of albumin, and thus represents a valuable model for the evaluation of the in vivo degradation of biodegradable NPs.

**Figure 4:**
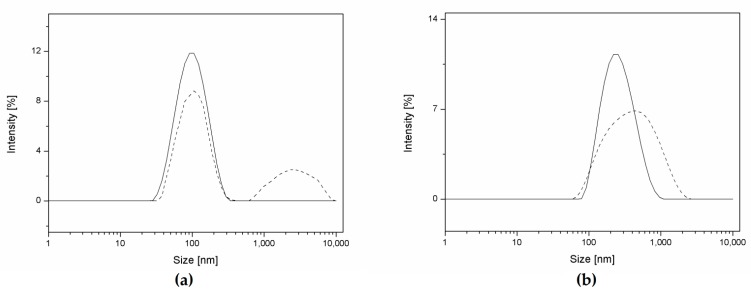
Evolution of the particle size distribution in cell medium during degradation. (**a**) Sample 20CL_3_ at 0 h (solid line) and after 48 h (dashed line). (**b**) Sample 20CL_3_PEG at 0 h (solid line) and after 216 h (dashed line).

In order to investigate the influence of the presence of PEG during the degradation study in cell medium, the particle size distribution (PSD) of two different samples (20CL_3_ and 20CL_3_PEG) are reported in Figure 4. At the beginning of the experiments, the PSD is both monodispersed and narrow, as already highlighted from the low PDI values listed in Table 2. After 48 h, the non-PEGylated sample shows the formation of micrometric aggregates due to the lower stabilization given by the Tween surfactant with respect to the covalently linked PEG chains present in the PEGylated samples (Figure 4a). In this case, the stabilizing power of Tween was not sufficient to avoid NP aggregation in this environment. This is probably due to the interaction between the proteins and adsorbed surfactant [39,40]. On the other hand, the PSD of the PEGylated sample becomes broader due to the degradation process, but does not highlight the presence of micrometric aggregates, even after 216 h of aging in cell medium (Figure 4b). Since most of the non-PEGylated samples highlighted the formation of big aggregates, both visually and from DLS, the stability and degradation studies were carried out only for the PEGylated samples. 

**Figure 5:**
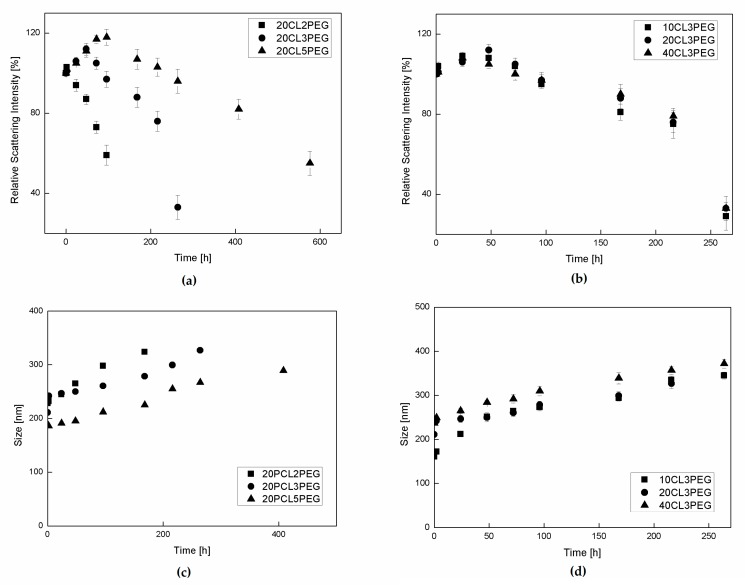
Evolution of relative scattering intensity and NP size of the samples in cellular medium. (**a**,**c**) Comparison between samples with different number of CL units in the side chains. (**b**,**d**) Comparison between samples with different DP of the backbone. The values and error bars represent the mean and standard deviation of three replicates.

Since the presence of proteins makes the quantitative evaluation of NP degradation in cell medium by the normally used techniques (SEC, weight loss, etc.) difficult, a qualitative study of NP degradation was performed by studying the evolution of their relative scattering intensity, which is a technique already used for studying the behavior of NPs in protein-containing media [23,28]. The scattering intensity is related to the NPs concentration, which is a function of both NP size and NP concentration. As the degradation proceeds, the water content of the NPs increases because of the increase in the polymer hydrophilicity. On the other hand, their polymer content decreases, since the degraded species derived from the hydrolysis of the CL chains leave the NPs. This process determines an initial increasing of the scattering light intensity, due to the initial swelling of the particles, and then its substantial lowering due to the particle degradation, as visible from Figure 5a,b. The cell medium environment is able to accelerate this process compared with PBS, since this protein-rich media increases the solubility of the oligomer chains released from the NPs. This way, the mass transfer of the oligomers from the NPs to the aqueous phase increases. In Figure 5a, the evolution of the relative scattering intensity is shown for samples 20CL_2_PEG, 20CL_3_PEG and 20CL_5_PEG, in order to investigate the influence of the side chain length on the NP degradation rate. For the sample 20CL_2_PEG, a significant decrease of the relative scattering intensity is visible after about 100 h, whereas a similar extent of degradation is visible after more than 200 h for the sample 20CL_3_PEG, and after more than 500 h for the sample 20CL_5_PEG. On the other hand, as shown in Figure 5b, the DP of the same HEMA-CL_3_ macromonomer does not affect the degradation rate of the NPs to a significant extent. These experiments show that in this system, the degradation rate is a function of the number of CL units *n* and is not a function of the DP of the polymer. A previous degradation study of HEMA-PCL polymers [41] highlighted higher degradation times compared with the ones obtained here. Either way, the polymer was not formulated in NPs in this case, but in powders, and the molar mass of the PCL side chains were higher, which confirms the trend found in this work. It is worth to note that since the number of CL units *n* for these materials is lower than the ones typically adopted for the more common biodegradable polymeric NPs, the degradation process is much faster than that of commercial PCL. Moreover, the obtained degradation time is comparable with the residence time of these NPs in the body [42], thus allowing the drug release to be mediated from the degradation of the NPs.

During the degradation the NPs undergo an increase in their size due to the swelling and degradation process as already reported. This trend is reported in Figure 5c,d where again the effect of the number *n* on the swelling behavior is visible. As *n* increases, the slope of the swelling process decreases due to the higher hydrophobicity and the lower swelling tendency. On the other hand the number *m* does not affect the rate of the NP size increasing. The samples were not purified from ethanol before dilution with PBS and cell medium since the concentration was low enough to not influence the NP behavior. In order to prove it a dialysis and a duplicate experiment was performed for the sample 20CL_2_PEG and reported in the Supplementary Materials (Table S1). 

**Figure 6:**
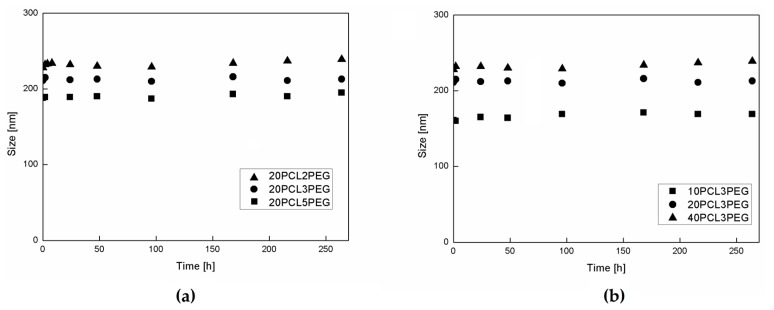
Evolution of NP size in phosphate-buffered saline solution (PBS). (**a**) Comparison between samples with different numbers of CL units in the side chains. (**b**) Comparison between samples with different DP of the backbone.

Another aqueous media used to study the stability and the degradation behavior of these NPs is isotonic PBS. This media is relevant since it is the one in which they are stocked, and it is also used for their intravenous injection. The particle size evolution of different NP formulations in PBS is presented in Figure 6. Since there is no significant variation in the NP size for all the samples even after 250 h, it is confirmed the effectiveness of the stabilization characteristics given by the PEG brushes which prevent the aggregation of the NPs in this environment. In addition it has been proved that the degradation in PBS is much slower being possibly to consider PBS as stock solution for these NPs. 

**Figure 7:**
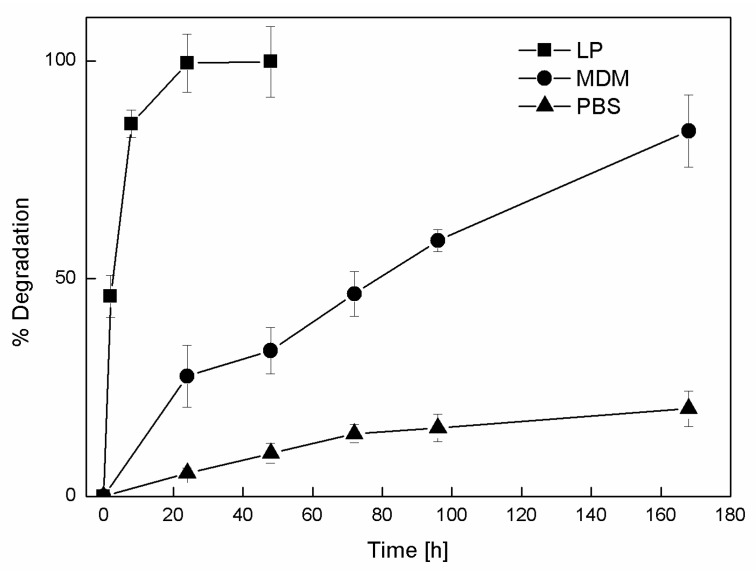
Evolution of NP degradation for the sample 20CL_3_PEG over time for three different media: Lipase solution (LP), cell medium (MDM), and PBS. The values and error bars represent the mean and standard deviation of three replicates.

A detailed degradation study was performed on the sample 20CL_3_PEG using HPLC to analyze the degradation products. To have a better understanding of the NP degradation process in vivo, the presence of enzymes also has to be taken into account. NPs with these sizes are rapidly endocytosed, and in this stage can undergo processes of enzymatic degradation. Cell medium contains proteins and nutrients such as blood plasma, but it does not contain enzymes, especially esterases, which are supposed to degrade biodegradable polyesters such as PCL. To look in this direction, a sample of 20CL_3_PEG was incubated with a solution of lipase. The degradation products were analyzed by HPLC-MS together with the ones produced in the incubation of the same sample in cell medium and PBS. The main degradation product consists of 6-hydroxycaproic acid (HCA), which was detected both by the UV and the MS detector. Traces of dimer and trimer were detected via MS, but via UV for all three media. Therefore, regardless of the fact that the three different conditions that have different degradation mechanisms, the degradation products are the same. The kinetic of the NP degradation in the three different media is shown in Figure 7.

The evolution of NP degradation shows a much faster kinetic when this is performed in the presence of lipase, where the NPs are totally degraded after 24 h. This confirms the effectiveness of the catalytic activity of this enzyme over the biodegradable chemical structure of the NPs. The biodegradability of the NPs is also sensitive to aqueous media that does not contain enzymes, such as medium and PBS, but as predicted from the previous DLS study, with different degradation kinetics. After seven days of incubation, more than 80% of HCA is released from the NPs exposed to cell medium, whereas just 20% is released from the ones exposed to PBS. These data also confirm the trend obtained from the DLS analysis for the cell medium incubation, since the degradation occurs in a similar amount of time. On the other hand, for the incubation of the NPs with PBS, the DLS analysis seems not able to predict the degradation that occurs, probably because of the lower extent of that for this case.

## 4. Conclusions

RAFT polymerization was applied to HEMA-CL*_n_* and PEGMA macromonomers to obtain biocompatible and biodegradable polymers for the production of NPs. The control over the polymerization process was verified by the linear correlation between polymer average molar mass and monomer conversion. The obtained polymers, characterized by GPC and ^1^H NMR, show *M*_w_/*M*_n_ lower than 1.2, pre-determinable molar mass and high monomer conversion. The structure of the comb-like polymer can be controlled in term of both backbone chain length, composition and side-chain length. Ethanol solutions of these polymers were then nanoprecipitated in water through a mixing device in order to produce NPs with tunable characteristics. The influence of the polymer structure on the NP properties was studied in terms of NP size, stability, and degradation behavior. These characteristics were found to be dependent upon both the nanoprecipitation process conditions and the polymer structure. The biocompatibility of the used materials, the characteristics of produced NPs, and the versatility of the production process make these materials interesting for employment in the biomedical field.

## Figures and Tables

**Table 1:** Characterizations for the first class (*m*CL*_n_)* and second class (*m*CL*_n_*PEG) of polymers.

Sample Name	*m*	*n*	*k*	χ	*M*_n_ th [Da]	*M*_n_ GPC [Da]	*M*_w_/*M*_n_
20CL_2_	20	2	-	0.956	7440	6862	1.14
10CL_3_	10	3	-	0.921	5000	7453	1.09
20CL_3_	20	3	-	0.983	9720	11,539	1.16
40CL_3_	40	3	-	0.952	19,160	18,641	1.12
20CL_5_	20	5	-	0.967	14,280	15,549	1.14
20CL_2_PEG	20	2	2	0.981	9440	8810	1.11
10CL_3_PEG	10	3	1	0.965	6000	7511	1.08
20CL_3_PEG	20	3	2	0.977	11,720	12,301	1.1
40CL_3_PEG	40	3	4	0.96	23,160	20,509	1.13
20CL5PEG	20	5	2	0.941	16,280	16527	1.09

**Table 2:** Characteristics of the nanoparticles (NPs) produced via nanoprecipitation at a concentration of 0.1 wt %.

Polymer Sample	NP Size [nm]	PDI
10CL_3_	85	0.114
20CL_2_	89	0.121
20CL_3_	72	0.106
20CL_5_	63	0.073
40CL_3_	63	0.090
20CL_2_PEG	228	0.142
10CL_3_PEG	161	0.101
20CL_3_PEG	211	0.122
40CL_3_PEG	237	0.145
20CL_5_PEG	188	0.089

## Supplementary Materials

Supplementary Materials are available online at www.mdpi.com/2073-4360/9/9/389/s1.
