# Supplementary material for: Synthesis and Nanoprecipitation of HEMA-CLn Based Polymers for the Production of Biodegradable Nanoparticles
Source: Polymers (Basel). 2017 Aug 23;9(9):389. doi: 10.3390/polym9090389 (PMC6418799; doi:10.3390/polym9090389)
Supplement: Supplementary file 1 [file polymers-09-00389-s001.pdf]

Supporting Information: Synthesis and nanoprecipitation of poly(ε-caprolactone) based comb-like polymers with well-defined structure

Simone Gatti, Azzurra Agostini, Raffaele Ferrari and Davide Moscatelli

1. NMR spectra

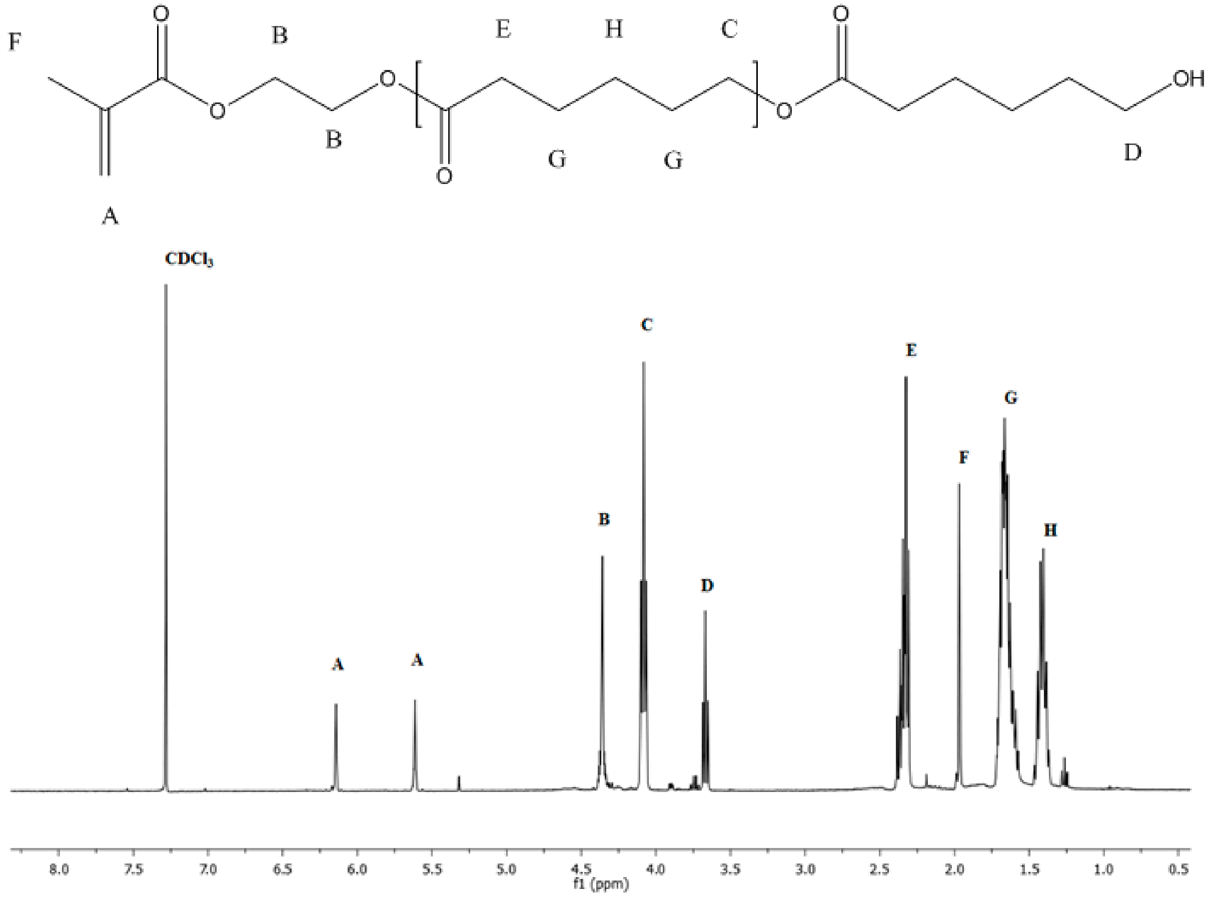

Figure S1. Structure and ^{1}H-NMR spectrum of HEMA-CL_3. The molecular weight of the produced macromonomers were evaluated by the formula $\mathrm{Mn} = \mathrm{Mn\_HEMA} + \mathrm{Mn\_CL} * (\frac{C}{D} + 1)$ where C and D are the integrals of the respective peaks

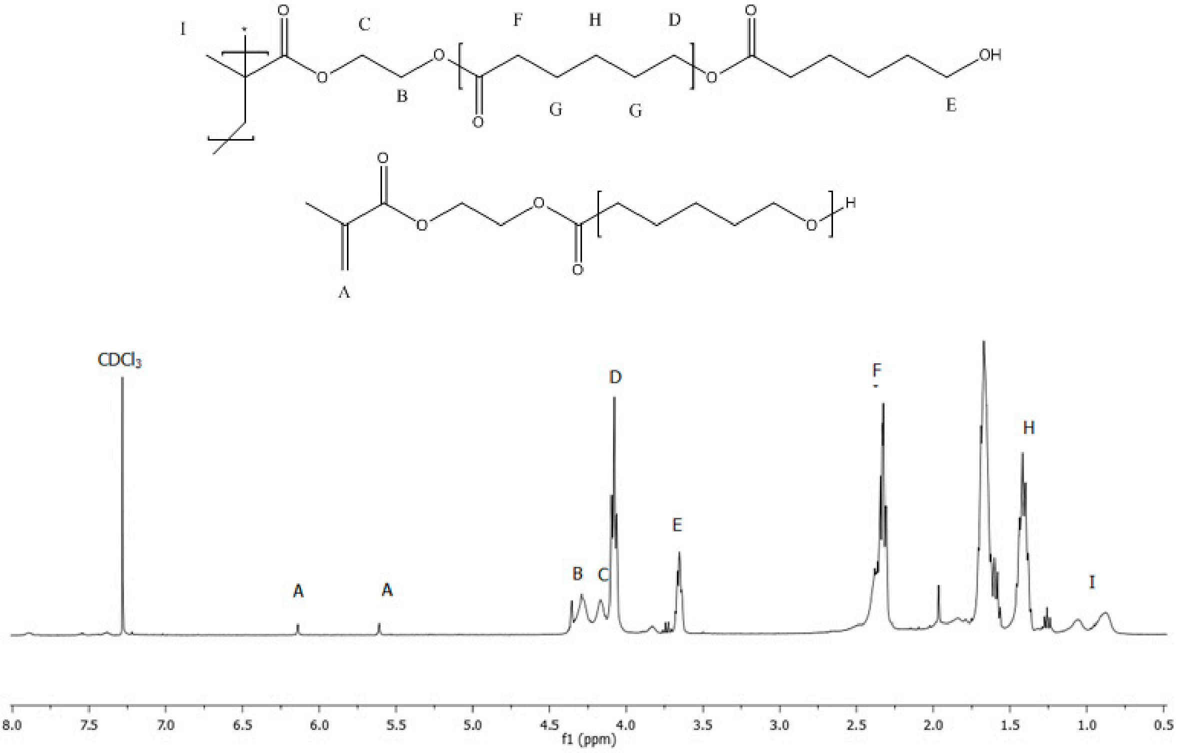

Figure S2. Structure and ^{1}H-NMR spectrum of the sample 20PCL3. The monomer conversions of the polymerizations were evaluated by the formula $\chi = 1-\frac{2A}{E}$ where A and E are the integrals of the respective peaks

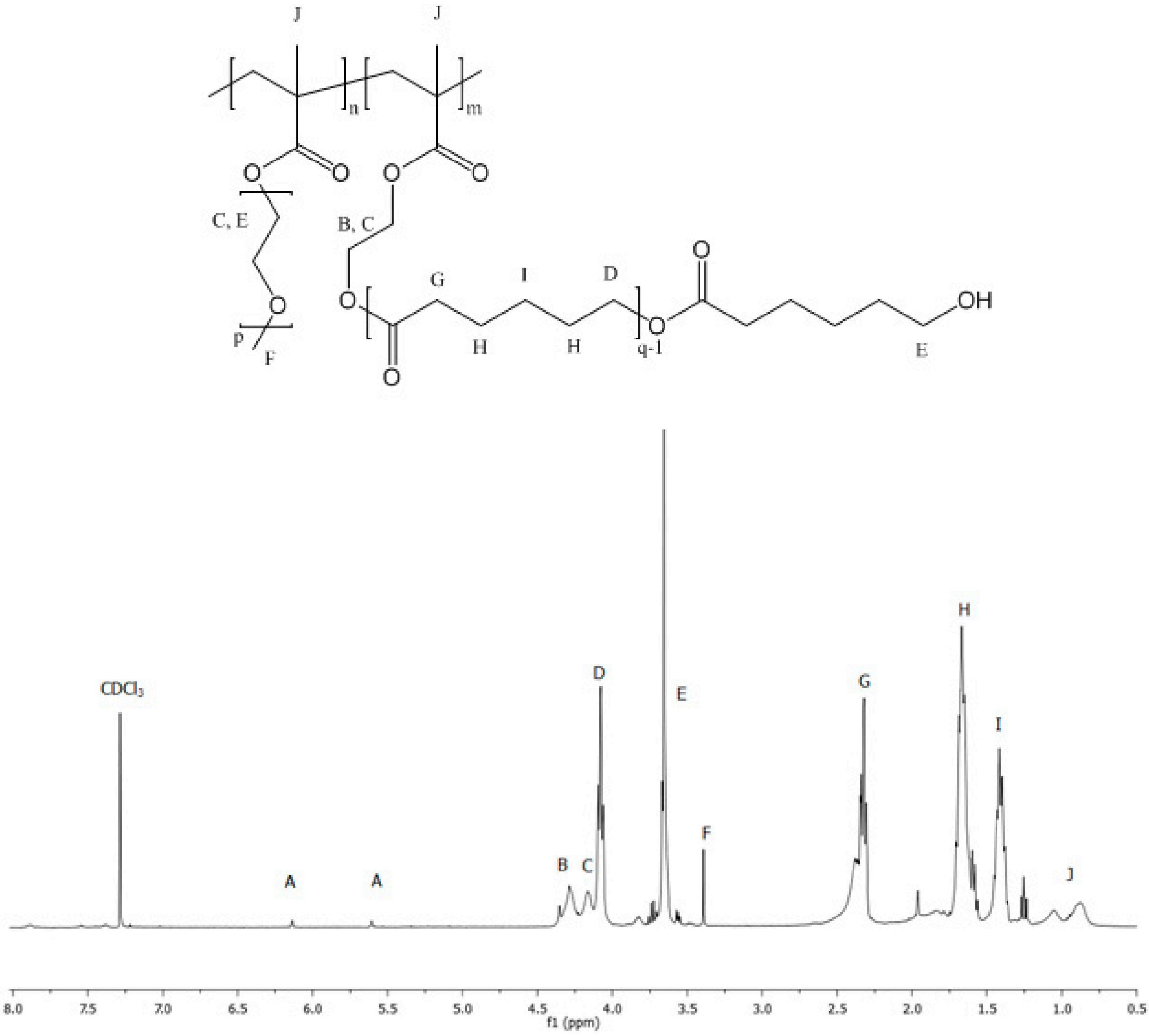

Figure S3. Structure and ^{1}H-NMR spectrum of the sample 20PCL3PEG. The monomer conversions of the polymerizations were evaluated by the formula $\chi = 1 - \frac{A}{F/3 + D/2(q-1)}$ where q is the number of CL units in the HEMA-CLq macromonomer (n in the Manuscript), A, F and D are the integrals of the respective peaks

2. Degradation study of 20PCL2PEG after dialysis

For the sample 20PCL2PEG a duplicate of the stability and degradation experiment was done after purification via dialysis. After the synthesis the obtained NP suspension was dialyzed using Spectra/Por dialysis membranes (MWCO 3500 Da) against distilled water for 4 h in order to remove Ethanol. The dialyzed suspension (1 mL) was diluted with cell medium (1 mL) and the relative scattering intensity was monitored by DLS. The comparison with the relative scattering intensity of the non-dialyzed sample is shown in Table S1.

Table S1. Evolution of relative scattering intensity

Time [h]	Dialyzed sample [%]	Non-dialyzed sample [%]
0	100	100
24	89	94
48	72	87
72	62	59
